# Supplementary material for: Mechanisms of Immune-Related Long Non-Coding RNAs in Spleens of Mice Vaccinated with 23-Valent Pneumococcal Polysaccharide Vaccine (PPV23)
Source: Vaccines (Basel). 2023 Feb 23;11(3):529. doi: 10.3390/vaccines11030529 (PMC10058596; doi:10.3390/vaccines11030529)
Supplement: Supplementary file 1 [file vaccines-11-00529-s001.zip › Supplementary Files/Supplementary Figures/English version of Supplementary Figures files.pdf]

Figure S1. Phenotypic data analysis of mice between control group and PPV23 treatment group (n=12). (A) Analysis of mice live weight during the experiment periods. (B) Serum immune factor concentration of IgG. (C) Serum immune factor concentration of IgM. (D) Serum immune factor concentration of IgA.

Figure S2. Genomic features of lncRNAs in mouse spleen. (A) The transcript length distribution of lncRNAs and mRNAs. (B) The exon number distribution of lncRNAs and mRNAs. (C) The ORFs length distribution of lncRNAs. (D) The ORFs length distribution of mRNAs.

Figure S3. The expression levels and amounts of lncRNAs and mRNAs. (A) Boxplots of lncRNAs and mRNAs expression levels (with log10 FPKM method) in the control group and treatment group. (B) The numbers of lncRNAs and mRNAs in mouse spleen in the control group and treatment group.

Figure S4. Volcano plot of the differential expression of mRNAs and lncRNAs in mouse spleen between control group and treatment group. (A) Differential expression of mRNAs. The blue points denote significantly down-regulated mRNAs, while the red points denote significantly up-regulated mRNAs. (B) Differential expression of lncRNAs. The blue points denote significantly down -regulated lncRNAs, while the red points denote significantly up-regulated lncRNAs.

Figure S5. GO and KEGG analysis of differentially mRNAs expression. (A) Histogram of GO enrichment of DE mRNAs. (B)Scatter plot of GO enrichment for

DE mRNAs. (C) Scatter plot of KEGG enrichment for DE mRNAs.

Figure S6. GO and KEGG analysis of differentially lncRNAs expression. (A) Histogram of GO enrichment of DE lncRNAs. (B)Scatter plot of GO enrichment for DE lncRNAs. (C) Scatter plot of KEGG enrichment for DE lncRNAs.

Figure S7. The Validation of RNA-seq by qRT-PCR (n=3). (A) qRT-PCR validation of six mRNAs. (B) qRT-PCR validation of six lncRNAs.

Figure S8. Relative mRNA expression levels of mouse Trim35 gene in spleen between PPV23 treatment group and the control group (n=3). ** indicate significant differences (P<0.01).
