# Supplementary figures and images for: Mechanisms of Immune-Related Long Non-Coding RNAs in Spleens of Mice Vaccinated with 23-Valent Pneumococcal Polysaccharide Vaccine (PPV23)
Source: Vaccines (Basel). 2023 Feb 23;11(3):529. doi: 10.3390/vaccines11030529 (PMC10058596; doi:10.3390/vaccines11030529)

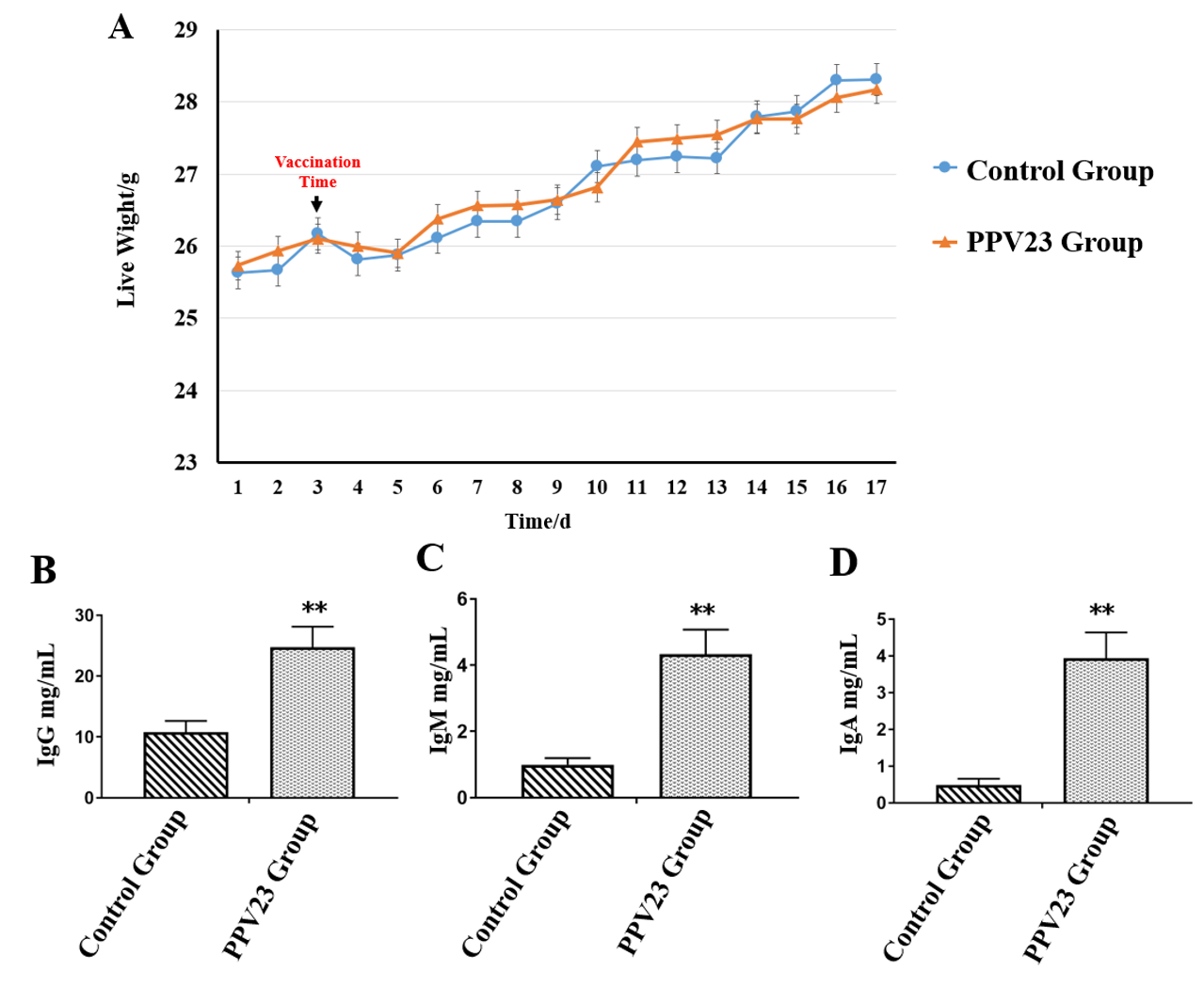

Supplement: Supplementary file 1 [file vaccines-11-00529-s001.zip › Supplementary Files/Supplementary Figures/Figure S1.png]

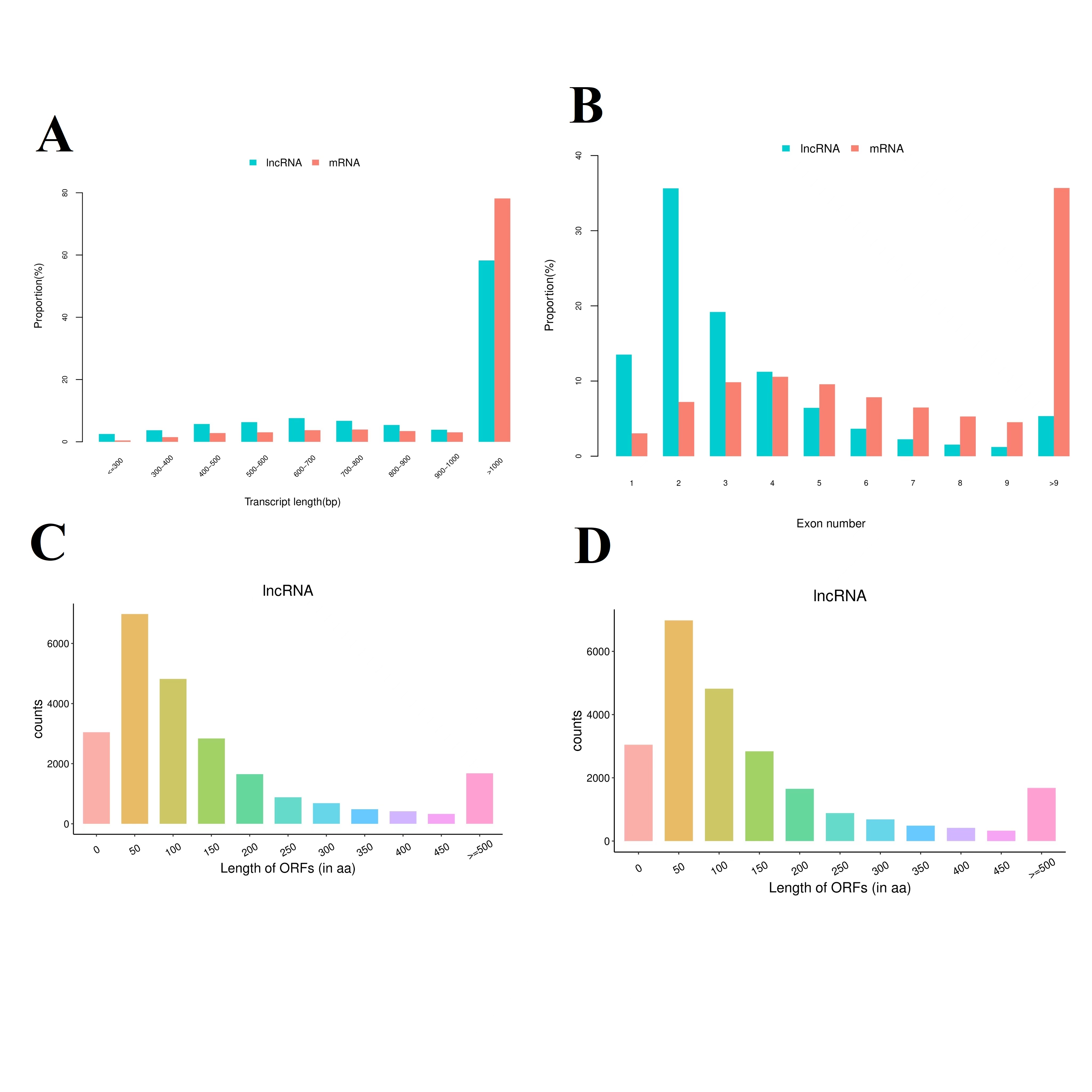

Supplement: Supplementary file 1 [file vaccines-11-00529-s001.zip › Supplementary Files/Supplementary Figures/Figure S2.jpg]

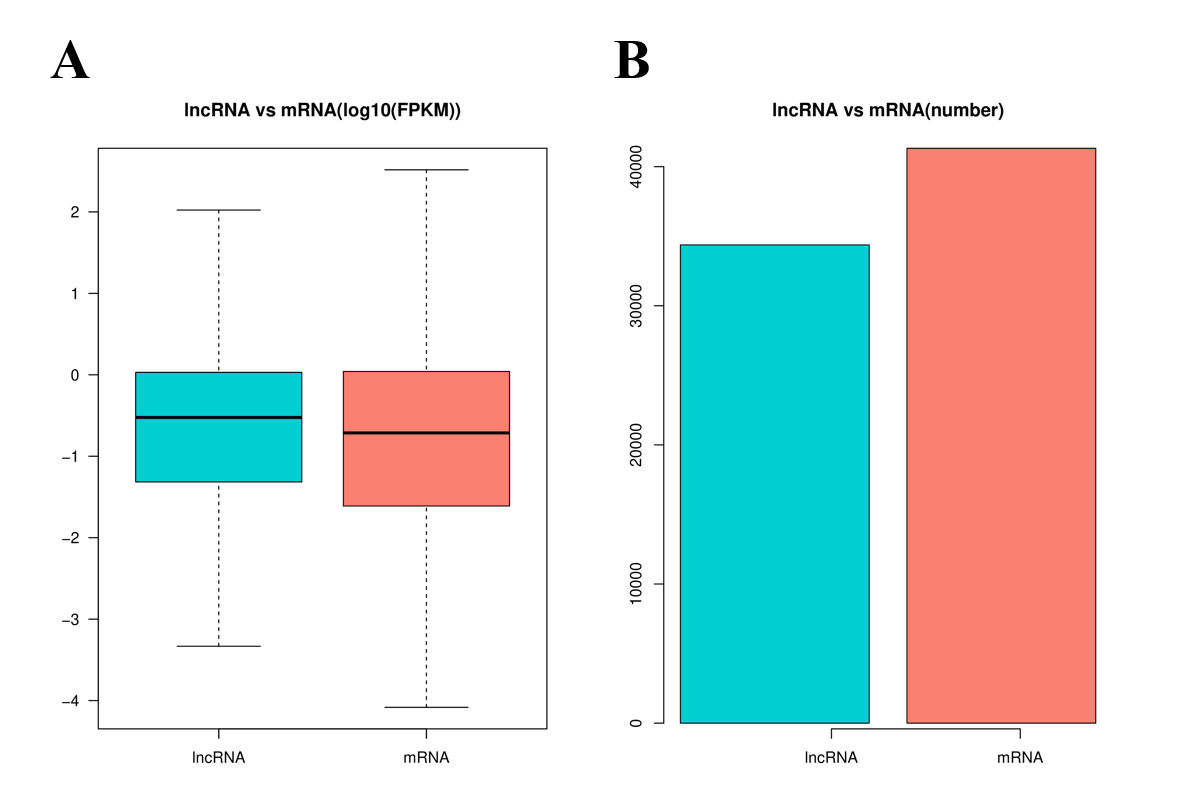

Supplement: Supplementary file 1 [file vaccines-11-00529-s001.zip › Supplementary Files/Supplementary Figures/Figure S3.png]

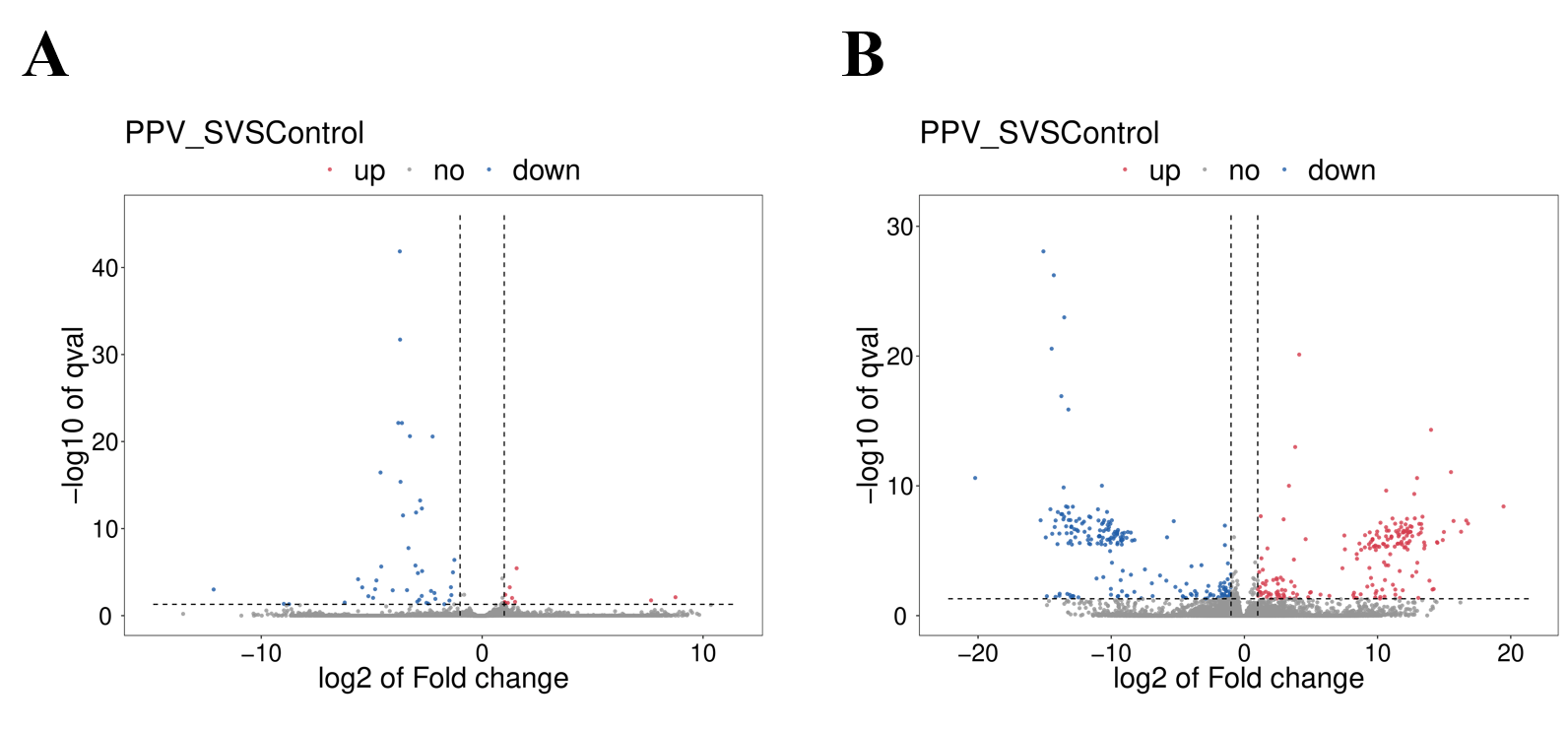

Supplement: Supplementary file 1 [file vaccines-11-00529-s001.zip › Supplementary Files/Supplementary Figures/Figure S4.png]

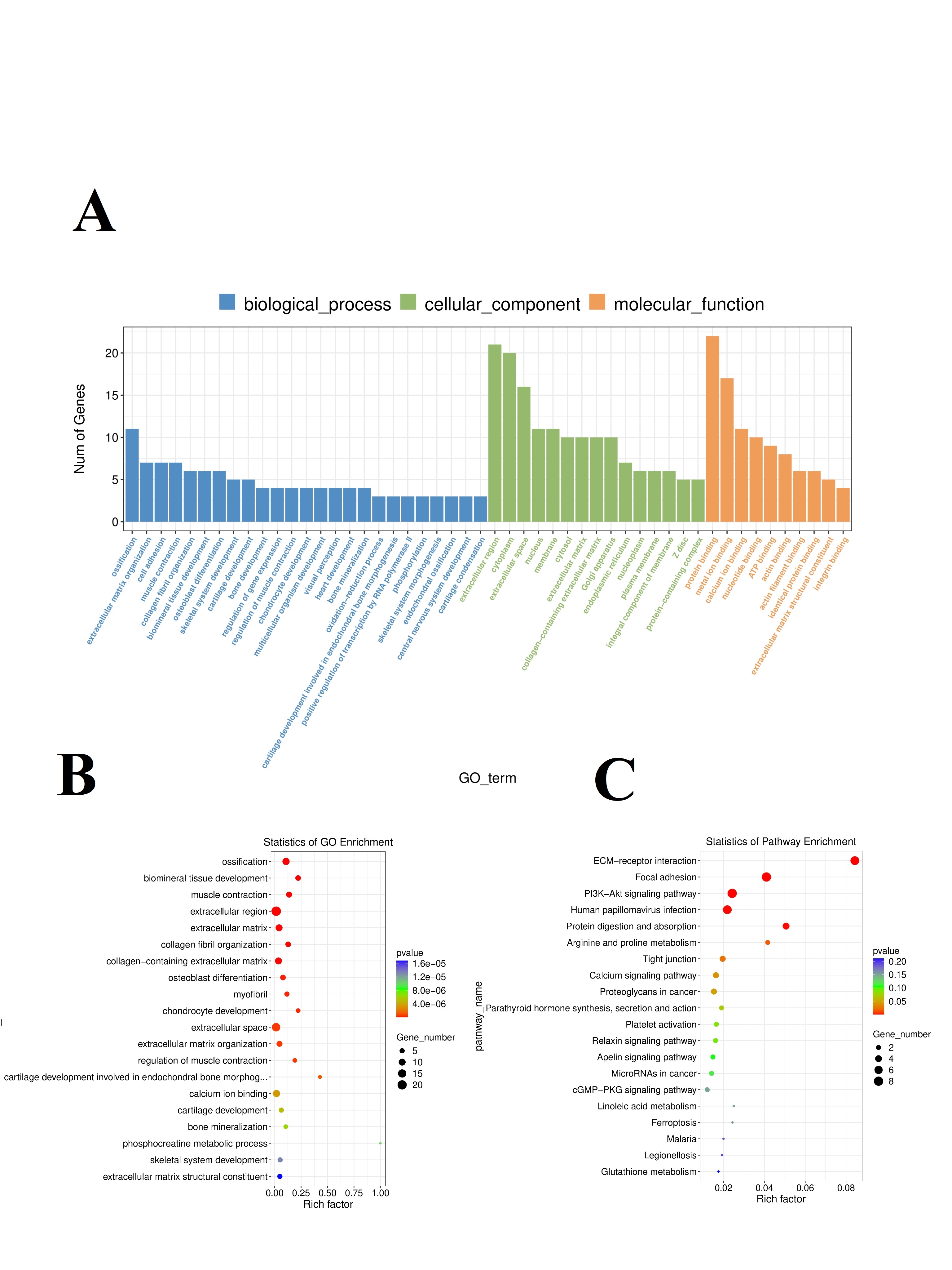

Supplement: Supplementary file 1 [file vaccines-11-00529-s001.zip › Supplementary Files/Supplementary Figures/Figure S5.jpg]

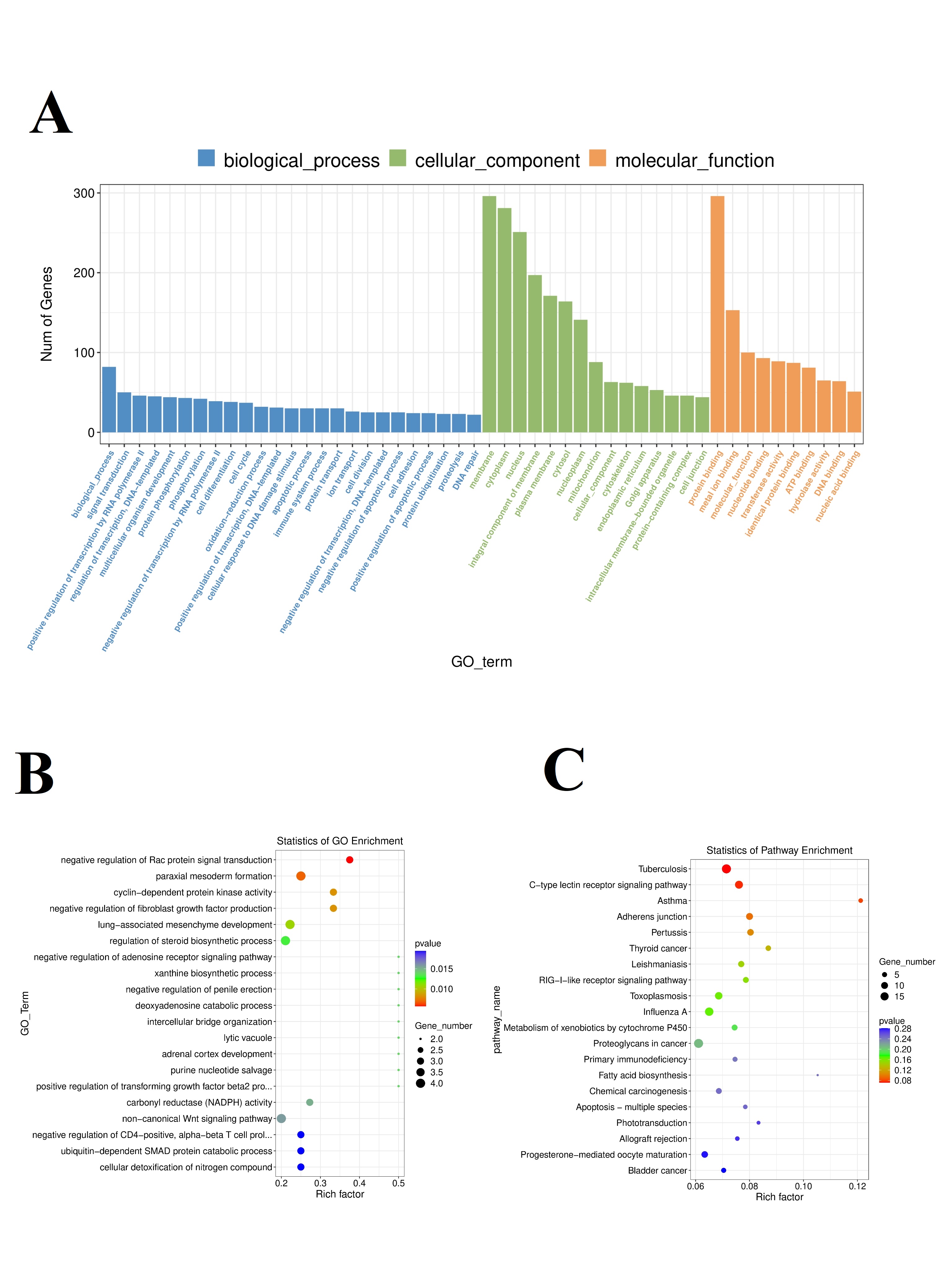

Supplement: Supplementary file 1 [file vaccines-11-00529-s001.zip › Supplementary Files/Supplementary Figures/Figure S6.jpg]

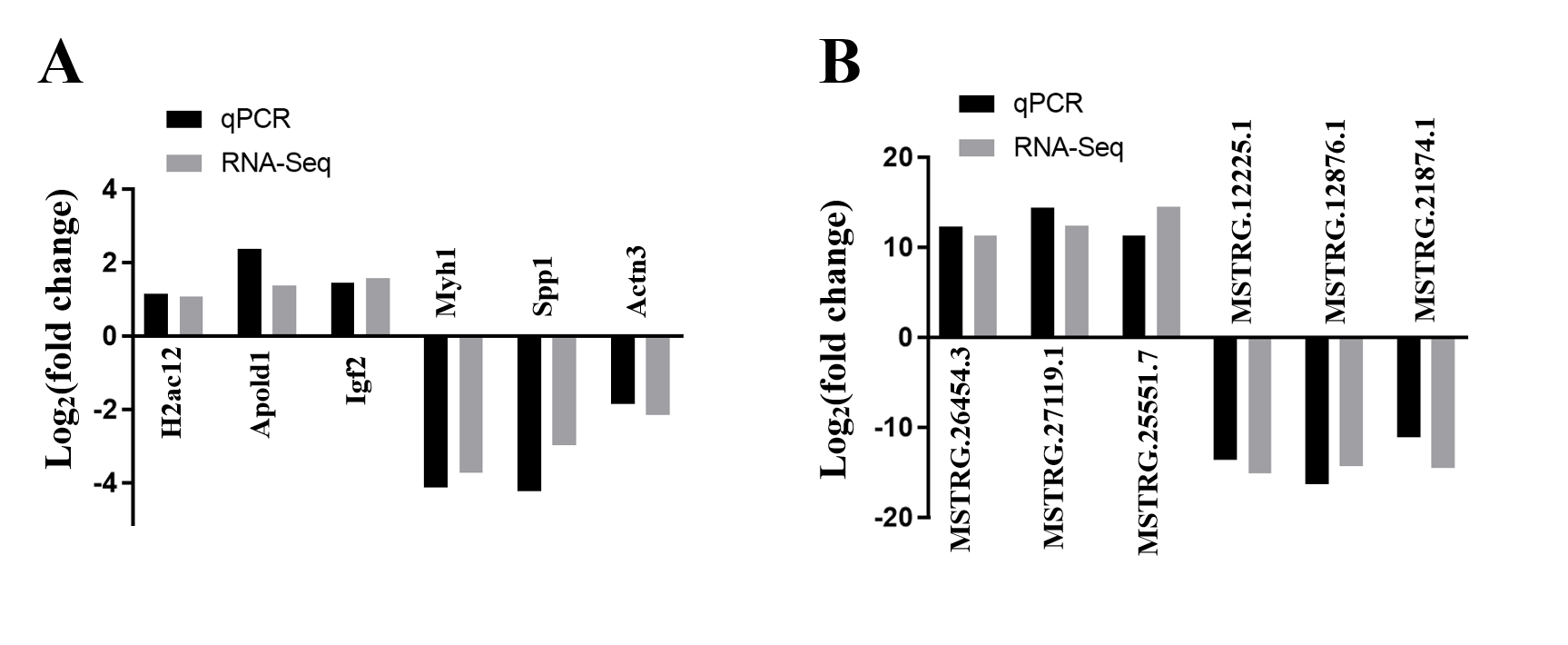

Supplement: Supplementary file 1 [file vaccines-11-00529-s001.zip › Supplementary Files/Supplementary Figures/Figure S7.png]

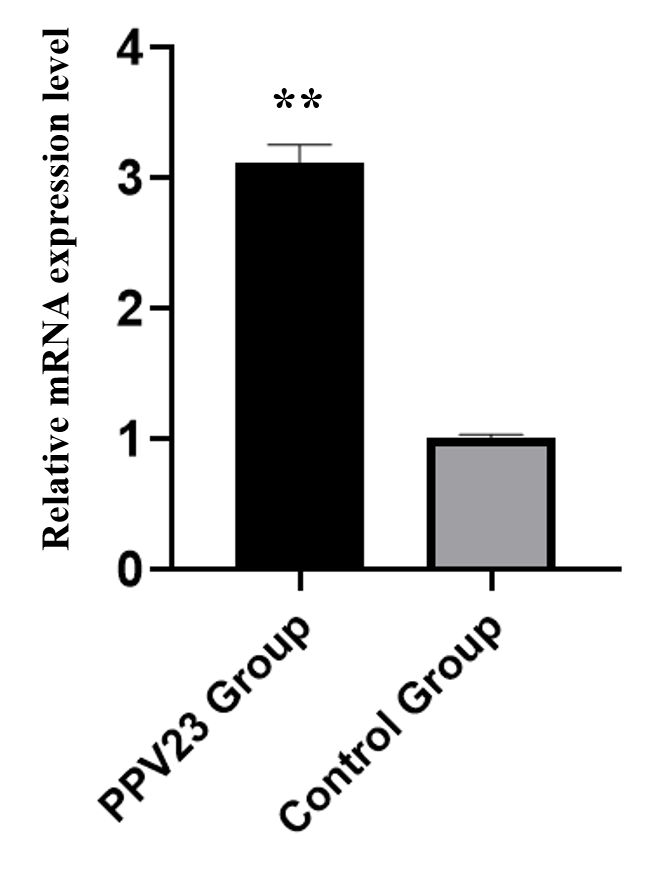

Supplement: Supplementary file 1 [file vaccines-11-00529-s001.zip › Supplementary Files/Supplementary Figures/Figure S8.png]
